# Retraction: Acute Omental Infarction Mimicking Acute Appendicitis

**DOI:** 10.7759/cureus.r81

**Published:** 2024-01-25

**Authors:** Rahaf J Owedah, Omar A Alshehri, Nourah I Alfneekh, Aishah H Alasmari, Dina W Hafiz, Yasamiyan A Alburayh, Mohammed A Alabdullah, Abdullah A Altarteer, Muhannad F Alharbi, Maram F Almutairi, Shahad S Aljohani, Ibtsam S Boudal, Malak A Alshammari

**Affiliations:** 1 Medicine, Al-Rayan Colleges, Medina, SAU; 2 Vascular Surgery, Asir Central Hospital, Abha, SAU; 3 Medicine, Qassim University, Qassim, SAU; 4 Ophthalmology, Asir Central Hospital, Abha, SAU; 5 Medicine, King Abdulaziz University, Jeddah, SAU; 6 Medicine, King Faisal University, Al-Ahsa, SAU; 7 Medicine, Wroclaw Medical University, Wroclaw, POL; 8 Medicine, University of Hail, Hail, SAU; 9 Family Medicine, Ministry of National Guard - Health Affairs, Riyadh, SAU; 10 Medicine, Imam Abdulrahman Bin Faisal University, Dammam, SAU

The Editors-in-Chief have retracted this article. Concerns were raised regarding the identity of the authors on this article. Specifically, Faisal Alhaway and Malak Shammari have stated that they were added to this article without their knowledge or approval. The identity of the other authors could also not be verified. As the appropriate authorship of this work cannot be established, the Editors-in-Chief no longer have confidence in the results and conclusions of this article.

